# Improvement and Maintenance of Clinical Outcomes in a Digital Mental Health Platform: Findings From a Longitudinal Observational Real-World Study

**DOI:** 10.2196/48298

**Published:** 2024-06-24

**Authors:** Lydia G Roos, Sara J Sagui-Henson, Cynthia Castro Sweet, Camille E Welcome Chamberlain, Brooke J Smith

**Affiliations:** 1 Department of Psychiatry and Biobehavioral Sciences University of California, Los Angeles Los Angeles, CA United States; 2 School of Medicine Stanford University Stanford, CA United States; 3 EvolveWell Research Partners Cincinnati, OH United States; 4 Modern Health San Francisco, CA United States

**Keywords:** digital mental health, employee health, depression, anxiety, well-being, mobile phone

## Abstract

**Background:**

Digital mental health services are increasingly being provided by employers as health benefit programs that can improve access to and remove barriers to mental health care. Stratified care models, in particular, offer personalized care recommendations that can offer clinically effective interventions while conserving resources. Nonetheless, clinical evaluation is needed to understand their benefits for mental health and their use in a real-world setting.

**Objective:**

This study aimed to examine the changes in clinical outcomes (ie, depressive and anxiety symptoms and well-being) and to evaluate the use of stratified blended care among members of an employer-sponsored digital mental health benefit.

**Methods:**

In a large prospective observational study, we examined the changes in depressive symptoms (9-item Patient Health Questionnaire), anxiety symptoms (7-item Generalized Anxiety Disorder scale), and well-being (5-item World Health Organization Well-Being Index) for 3 months in 509 participants (mean age 33.9, SD 8.7 years; women: n=312, 61.3%; men: n=175, 34.4%; nonbinary: n=22, 4.3%) who were newly enrolled and engaged in care with an employer-sponsored digital mental health platform (Modern Health Inc). We also investigated the extent to which participants followed the recommendations provided to them through a stratified blended care model.

**Results:**

Participants with elevated baseline symptoms of depression and anxiety exhibited significant symptom improvements, with a 37% score improvement in depression and a 29% score improvement in anxiety (*P* values <.001). Participants with baseline scores indicative of poorer well-being also improved over the study period (90% score improvement; *P*=.002). Furthermore, over half exhibited clinical improvement or recovery for depressive symptoms (n=122, 65.2%), anxiety symptoms (n=127, 59.1%), and low well-being (n=82, 64.6%). Among participants with mild or no baseline symptoms, we found high rates of maintenance for low depressive (n=297, 92.2%) and anxiety (n=255, 86.7%) symptoms and high well-being (n=344, 90.1%). In total, two-thirds of the participants (n=343, 67.4%) used their recommended care, 16.9% (n=86) intensified their care beyond their initial recommendation, and 15.7% (n=80) of participants underused care by not engaging with the highest level of care recommended to them.

**Conclusions:**

Participants with elevated baseline depressive or anxiety symptoms improved their mental health significantly from baseline to follow-up, and most participants without symptoms or with mild symptoms at baseline maintained their mental health over time. In addition, engagement patterns indicate that the stratified blended care model was efficient in matching individuals with the most effective and least costly care while also allowing them to self-determine their care and use combinations of services that best fit their needs. Overall, the results of this study support the clinical effectiveness of the platform for improving and preserving mental health and support the utility and effectiveness of stratified blended care models to improve access to and use of digitally delivered mental health services.

## Introduction

### Background

The prevalence of mental health needs in the United States has been on an upward trend in recent years, with 21% of adults meeting criteria for a mental, behavioral, or emotional disorder in 2020, up from 18% in 2010 [1]. National data indicate that <50% of people with mental health concerns are able to access mental health services [1]. Traditional models of mental health care are inadequate, as they rely heavily on high-cost providers delivering scheduled, time-limited encounters, and training programs are decades away from adequately closing the provider shortage gaps [2,3]. In addition to a pervasive shortage of mental health professionals to provide needed care [4], issues related to cost, accessibility, and stigma also prevent individuals from accessing evidence-based care to address mental health concerns [1]. Thus, innovative models for mental health care that are scalable, resource sensitive, and acceptable to individuals are needed to sufficiently improve the provision of robust mental health care in the United States.

Innovative and flexible models of mental health care leverage technology and telecommunications to provide more rapid and scalable access to a myriad of mental health services, from self-guided “self-help” techniques to access to providers who deliver secure, remote care [5]. An advantageous feature of digital mental health platforms is their flexibility in offering a variety of care modalities, enabling users to exercise their preferences in accessing care in a way that best fits their needs and comfort level. Stepped care delivery models further accelerate improvements in mental health care access and affordability. There are currently 2 models: progressive and stratified. A progressive model recommends the lowest-intensity intervention first to all individuals and intensifies care if or when symptoms do not improve [6]. This is the prevailing system used by the United Kingdom National Health Service’s Talking Therapies program (formerly called Improving Access to Psychological Therapies) [7]. The evidence supporting this model suggests that patients’ baseline symptomatology does not impact the efficacy of low-intensity or high-intensity treatments [8,9].

However, recent research suggests the Improving Access to Psychological Therapies program may not adequately support or match the complexity of patients’ presenting mental health issues [10]. There are several criticisms [11] of the progressive approach, including (1) low-intensity interventions may not be suitable or acceptable for everyone; (2) patients who do not respond to low-intensity treatment may develop negative attitudes toward treatment or be deterred from undergoing further treatment; (3) engaging in high-intensity treatment after a minimal intervention may be unnecessarily burdensome; and (4) those with greater clinical needs may have to wait longer to receive a more effective level of care and, in the interim, experience an exacerbation of symptoms and additional impairment.

As an alternative, a *stratified* model considers patient characteristics, preferences, and baseline mental health symptoms to identify and deliver the most clinically effective yet least burdensome and least costly initial intervention from a range of care modalities of different intensities [12]. Stratifying care with personalized recommendations is thought to be more patient-centered and is responsive to key drawbacks of the progressive stepped care approach [11]. In some stratified systems, more specifically blended care models, patients can access multiple modalities simultaneously; that is, they can use their recommended treatment modality as well as additional modalities of lower intensity than their recommended starting point (eg, digital tools plus provider sessions, as opposed to digital-only or provider-only session). While advantageous from a delivery perspective and found to be effective [13,14], stratified, blended models can be more difficult to evaluate because of the complexity and variety of care pathways offered to patients and the variability of “blends” that patients may use at different points in time.

### Research Questions

Prior findings regarding the clinical superiority of progressive models over stratified models are mixed [15,16]. These mixed results and the criticisms of progressive stepped care suggest that by incorporating patient-level factors to match individuals with the most effective yet least costly mental health services, stratified, blended models can offer more personalized care and increase access while optimizing resources. In this study, we examined the effectiveness of and engagement in a digital mental health platform that uses a stratified blended care approach to deliver therapy, coaching, and self-guided digital services. Specifically, we tested two research questions: (1) Was this approach clinically effective, that is, did participants with elevated baseline mental health symptoms significantly improve their mental health as defined by depression, anxiety, and well-being scores, and did participants without elevated baseline symptoms maintain good mental health from baseline to 3-month follow-up? (2) Was this approach effective in stratifying resources, that is, did people follow, underuse, or overuse mental health services at the levels they were recommended?

## Methods

### Design and Participants

This investigation was conducted as part of a larger prospective, observational study of individuals who received services through an employer-sponsored digital mental health benefits platform (Modern Health Inc). The study time frame was September 20, 2021, through May 31, 2022. Participants were eligible if they were aged ≥18 years; were based in the United States; were onboarded with the employer-sponsored mental health benefit; had access to a smartphone, a tablet, or a computer; and had engaged with at least 1 piece of digital content or matched with a coach or therapist (see the Intervention section for more detailed descriptions of the services).

### Ethical Considerations

This study protocol was reviewed and approved by the Western Clinical Group Institutional Review Board (protocol no 1316167). Participants provided informed consent to participate in this investigation. The Western Clinical Group Institutional Review Board authorized a waiver of documentation of consent for the team to collect consent through secure electronic methods.

All data were deidentified for the purpose of analyses. Participants were compensated with a US $25 digital gift card upon completion of each of the 3 surveys in this investigation.

### Procedures

**Figure 1:**
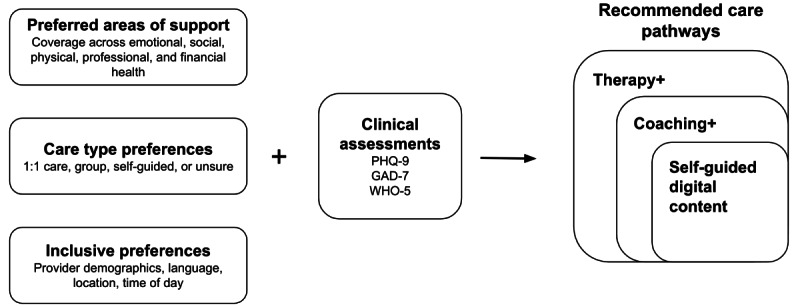
Stratified blended care model incorporating care preferences and clinical assessments into personalized care recommendations. GAD-7: 7-item Generalized Anxiety Disorder scale; PHQ-9: 9-item Patient Health Questionnaire; WHO-5: 5-item World Health Organization Well-Being Index.

Participants registered for an account through a mobile app or a website and completed onboarding assessments, including questions designed to assess participants’ areas of focus and care modality preferences, as well as validated measures to assess depressive and anxiety symptoms and well-being (described in the Study Measures section). A proprietary algorithm factored in a combination of the participant’s clinical acuity, their modality preference, and their topic of focus to recommend an initial care pathway (eg, digital programs, coaching, and therapy). Participants were not required to follow the recommendation; instead, it was offered as an appropriate suggested starting point. Participants who were recommended therapy also had access to coaching and digital content, and those who were recommended coaching also had access to digital content as part of their recommendations (Figure 1). Participants could also self-refer or be referred by a provider to a different combination of care from their recommended combinations.

Eligible members were invited to complete a screener for the study via email, which collected their demographic information (age, gender identity, race, and ethnicity). All screeners were sent within 2 weeks of onboarding, with most members receiving the screener approximately 1 week after onboarding. During this time, the members were able to engage with the digital mental health services outlined in the Intervention section. A total of 2 factors determined the length of time it took to send the screener: research staff availability and demographic balancing. Limits were set such that enrolled study participants reflected the current distribution of age, gender, ethnic/racial identity, and mental health symptom acuity observed in the platform’s commercial population. Out of the 8786 individuals who were eligible and invited to participate, 950 (10.81%) enrolled, provided informed consent, and completed the baseline survey, hosted by Qualtrics (**Qualtrics** International Inc). They were then emailed a link to complete a follow-up survey 12 weeks after the baseline survey. The baseline and follow-up surveys each took 30 to 45 minutes to complete.

### Intervention

#### Digital Health Services

Participants could engage in all the following digital mental health services. All services were paid for by the participant’s employer, at no cost to the individual.

#### Telecoaching and Teletherapy

Coaches certified by the International Coaching Federation accredited program provided telecoaching services, and therapists who were licensed and had an advanced degree in clinical psychology or a related field (eg, PhD, PsyD, licensed clinical social worker, licensed marriage and family therapist, or licensed professional counselor) provided teletherapy services to participants. All visits were conducted via a secured videoconferencing platform. Participants could also communicate with their therapist or coach through in-app messaging. All coaches had at least 150 hours of coaching experience, were vetted by a provider management team to ensure their work aligned with evidence-based practices, and completed an additional 6 hours of training from the company clinical strategy team in evidence-based techniques (eg, cognitive behavioral approaches) and culturally centered care. Coaches were also vetted and trained on how to assess for high-risk situations that may require a participant’s referral to a therapist or crisis resource.

Therapists were selected for their use of evidence-based practices, such as cognitive behavioral therapy and acceptance and commitment therapy. All coaches and therapists were trained on the company’s proprietary model of care. The number of therapy and coaching sessions attended by participants depended on the number of sessions covered by their employer, as well as on personal preferences and their level of need.

#### Self-Guided Digital Content

All participants had unlimited access to a digital library of mental health programs and resources that they could access at any time. These included short (2 minutes each) daily exercises; interactive programs and podcasts (2 to 15 minutes each); mindfulness exercises such as meditations and breathing exercises (2 to 15 minutes each); and self-paced structured educational lessons (several chapters of content, akin to self-help workbooks, that are paced to be completed over several weeks). Digital programs were developed and designed by an in-house team of clinical psychologists and covered topics such as emotions, relationships, professional life, healthy lifestyles, and finances. Engagement across all digital resources was combined in analyses to represent total digital program engagement.

### Study Measures

#### Demographic Information

Participants self-reported demographic characteristics such as age; gender identity (to select all that applied from a list: agender, genderqueer or genderfluid, Māhū [third gender], man, muxe, nonbinary, questioning or unsure, 2-spirit, woman, prefer to self-describe, and prefer not to say); and race and ethnicity (to select all that applied from a list: American Indian or Alaska Native; Asian; Black or African American; Hawaiian or Pacific Islander; Hispanic, Latinx, or Spanish; White [not Hispanic or Latinx]; multiracial) during the screener. On the basis of participants’ demographics, 3 categories were used in analyses: men, women, and nonbinary (all other categories except “prefer to self-describe” or “prefer not to say” collapsed).

#### Depressive Symptoms

The 9-item Patient Health Questionnaire (PHQ-9) [17] was used to assess the presence of depression symptoms over the past 2 weeks at baseline and follow-up. Participants responded on a 4-point scale (0=“not at all” to 3=“nearly every day”). Possible ranges for scores include 0-27, with higher scores indicating a higher severity of depression symptomatology. The clinically validated cutoff for probable depression (“high risk”) is ≥10 [17], and clinical improvement was indicated when participants’ scores decreased by >6 points [18].

#### Anxiety Symptoms

The 7-item Generalized Anxiety Disorder Questionnaire (GAD-7) [19] was used to assess the presence of anxiety symptoms over the past 2 weeks at baseline and follow-up. Participants responded on a 4-point scale (0=“not at all” to 3=“nearly every day”). The possible range was 0-21, with higher scores indicating a higher severity of anxiety symptomatology. The clinical cutoff score for probable anxiety disorder (“high risk”) is ≥8 [20], and improvement was indicated when participants’ scores decreased by >4 points [21].

#### Well-Being

The 5-item World Health Organization Well-Being Index (WHO-5) [22] was used to assess well-being over the past 2 weeks at baseline and follow-up. Participants responded on a 6-point scale (0=“at no time” to 5=“all of the time”). Scores are summed and multiplied by 4, giving a total range of 0-100, with higher scores indicating greater subjective well-being. The clinical cutoff indicating low well-being (“high risk”) is ≤28; recovery was indicated when the baseline score was <28 and the 3-month score was >28. Prior research has defined clinical improvement in well-being as an increase of at least 10 points [22,23].

#### Platform Engagement

To operationalize care engagement, we categorized participants based on whether they (1) engaged in care at the level recommended, (2) underused care, or (3) overused care. We defined engaging or following the care recommendation as a participant using their level of recommended care (with the ability to use anything below that level of care). Overusing care occurred when participants used a higher level of care than what they were initially recommended (intensified their care above what was originally recommended, regardless of whether that use step-up was self-referred or referred by a provider). Because participants who were originally recommended therapy as their care modality could not step up their care any higher, only participants who were recommended coaching or digital content or coaching could overuse care. Finally, we defined the underuse of care as participants using a lower level of care than their recommendation and not using any higher care modality. Because participants who were recommended digital content could not use a lower level of care, only participants who were recommended therapy or coaching could underuse it.

### Statistical Analysis

Analysis of participant demographics and preliminary analyses were conducted using descriptive statistics and frequencies. We used McNemar *χ*^2^ tests and paired sample 2-tailed *t* tests to examine the clinical effectiveness of the platform, that is, mental health improvement, recovery, or maintenance. Specifically, we used McNemar *χ*^2^ tests to assess whether the percentage of participants categorized as high risk in each mental health outcome significantly decreased from baseline to follow-up. We used paired sample *t* tests to assess whether changes in each outcome (measured continuously) were significantly improved from baseline to follow-up.

We also reported the percentage of participants who reliably improved, recovered, and maintained their mental health. For participants who met the clinical cutoff for outcomes at baseline (“high risk”), we examined improvement or recovery in symptoms from baseline to the 3-month follow-up. Improvement in each measure was indicated when participants’ scores changed by established clinical thresholds (see the Study Measures section), and recovery in each measure was indicated when participants met or exceeded the clinical cutoff at baseline (ie, were categorized as “high risk”) but did not meet the clinical cutoff at follow-up (ie, were categorized as “low risk”). Maintenance was indicated when participants remained below the clinical cutoff from baseline (“low risk”) to follow-up. Continuous variables (ie, depressive and anxiety symptoms and well-being at baseline and follow-up) were examined for kurtosis and skewness; all values were between −1 and 1. Thus, original values were used in analyses.

We assessed our research question regarding use-care recommendations using frequencies and descriptive statistics. We report the percentage of participants who engaged in their recommended services, the percentage who overused services, and the percentage who underused services.

## Results

### Study Participants and Preliminary Analyses

A total of 950 members completed the baseline survey, of which 696 (73.2%) completed the follow-up survey. Of the 696 with full data, 528 (75.9%) members engaged with the platform (eg, used digital content, had teletherapy, or had a telecoaching visit) at least once between baseline and follow-up. There were 10 (n=528, 1.9%) participants who were not included in data analyses because they were recommended a care modality for which we did not have engagement data (ie, group psychoeducation sessions), and 9 (n=528, 1.7%) participants were not provided with a recommended care plan for reasons unknown. There were no significant baseline clinical differences between people who did not engage with anything on the Modern Health app after baseline (n=168) and those who did engage. As engagement with the app at least once during the analytic time frame was necessary for inclusion in the study, the remaining analyses included the final 509 (53.6% of enrolled) participants for every outcome except for anxiety, for which there were missing data, that yielded a total of 506 (53.2% of enrolled) for anxiety analyses.

The *t* test and *χ*^2^ analyses comparing baseline data from individuals who met final eligibility criteria (509/950, 53.6%) versus those who did not meet eligibility criteria (441/950, 46.4%) revealed no significant differences in age, gender identity, or race and ethnicity at baseline, and the groups were not significantly different on depression, anxiety, or well-being scores when assessed continuously (*P* values >.10). The participants who met the final eligibility criteria were significantly less likely to meet the clinical cutoffs for depressive and anxiety symptoms and low well-being (*P* values <.001).

The descriptive statistics of the sample are provided in Table 1.

Of the 509 participants, 342 (67.2%) engaged with digital content on the app at least once. A total of 159 (31.2%) participants attended at least 1 therapy visit, and 296 (58.2%) participants attended at least 1 coaching visit. The participants that attended therapy or coaching visits typically saw 1 provider over the course of the study (149/159, 93.7% and 283/296, 95.6%, respectively). The maximum number of therapists and coaches seen by any 1 participant were 3 and 2, respectively.

**Table 1:** Descriptive statistics of sample^a^ (n=509).

		Values	Participants at baseline, n (%)	Participants at 3-month follow-up, n (%)
Age (years), mean (SD)		33.9 (8.7)	—^b^	—
**Race/ethnicity, n (%)**				
	Asian	91 (17.9)	—	—
	American Indian or Alaska Native	1 (0.2)	—	—
	Black	33 (6.5)	—	—
	Hispanic, Latinx, or Spanish origin	44 (8.6)	—	—
	White (non-Hispanic or Latinx)	305 (59.9)	—	—
	Multiracial	33 (6.5)	—	—
**Gender identity, n (%)**				
	Women	312 (61.3)	—	—
	Men	175 (34.4)	—	—
	Nonbinary	22 (4.3)	—	—
**Scores^c^**				
	Depressive symptoms above clinical cutoff	—	187 (36.7)	106 (20.8)
	Anxiety symptoms above clinical cutoff	—	215 (42.2)	156 (30.8)
	Well-being below clinical cutoff	—	127 (25)	94 (18.5)

^a^n=509 for depressive symptoms and well-being and n=506 for anxiety symptoms.

^b^—: not available.

^c^Possible ranges for scores include 0 to 27 for depressive symptoms, 0-21 for anxiety symptoms, and 0-100 for well-being. Clinical cutoffs at baseline were ≥10 for depressive symptoms, ≥8 for anxiety symptoms, and ≤28 for well-being.

### Improvement and Recovery in Mental Health Symptoms Among Participants at Higher Risk at Baseline

All improvement, recovery, and change in mental health results among participants who met the clinical cutoff on each measure at baseline are presented in Tables 2 and 3.

Participants at a higher risk for depressive symptoms at baseline reported a statistically significant 37% improvement in PHQ-9 scores at follow-up, on average, with 65.2% (122/187) experiencing clinically meaningful improvement or recovery (*P*<.001). Participants at a higher risk for anxiety symptoms at baseline reported a statistically significant 29% improvement in GAD-7 scores at follow-up, on average, with 59.1% (127/215) experiencing clinically meaningful improvement or recovery (*P*<.001). Participants at a higher risk for lower well-being at baseline reported a statistically significant 90% improvement in WHO-5 scores at follow-up, on average, with 65.6% (82/127) experiencing clinically meaningful improvement or recovery (*P*=.002). Post hoc sensitivity analyses revealed that our models among higher-risk participants were sensitive to detect small effect sizes (Cohen *d_z_*=0.20 for depressive symptoms, 0.19 for anxiety symptoms, and 0.25 for well-being), with 80% power and α=.05.

**Table 2:** Clinical improvement, recovery, and change in mental health from baseline to follow-up among participants at higher risk at baseline (n=509).

Baseline symptoms^a^	Improvement, n (%)	Recovery, n (%)	Improvement and recovery, n (%)	Improvement or recovery, n (%)
Depressive symptoms (n=187)	83 (44.4)	106 (56.7)	67 (35.8)	122 (65.2)
Anxiety symptoms (n=215)	107 (49.8)	98 (45.6)	78 (36.3)	127 (59.1)
Well-being (n=127)	76 (59.8)	71 (55.9)	65 (51.2)	82 (64.6)

^a^Possible ranges for scores include 0-27 for depressive symptoms, 0-21 for anxiety symptoms, and 0-100 for well-being.

**Table 3:** Change in mental health from baseline to follow-up among participants at higher risk at baseline (n=509).

Baseline symptoms^a^	Baseline, mean (SD)^b^	Follow-up, mean (SD)^c^	Cohen *d*	*t* test (*df*)	*P* value
Depressive symptoms (n=187)	14.13 (3.43)	8.91 (4.25)	1.16	15.81 (186)	<.001
Anxiety symptoms (n=215)	12.68 (3.69)	8.99 (4.76)	0.75	11.02 (214)	<.001
Well-being (n=127)	20.44 (6.80)	38.80 (19.75)	−0.93	−10.50 (126)	<.001

^a^Possible ranges for scores include 0-27 for depressive symptoms, 0-21 for anxiety symptoms, and 0-100 for well-being.

^b^Improvement was indicated when depressive and anxiety symptom scores decreased by ≥6 points, and ≥4 points, respectively, and when well-being scores increased by ≥10 points.

^c^Recovery was indicated when participants met or exceeded the clinical cutoff at baseline, but not at follow-up.

### Maintenance of Mental Health Symptoms Among Participants at Lower Risk at Baseline

All maintenance and change in mental health results among participants who had mild or no symptoms on each measure at baseline are presented in Table 4.

Participants at a lower risk for depressive symptoms at baseline reported a small, significant improvement in PHQ-9 scores at follow-up, on average, with 92.2% (297/322) maintaining their low symptom status. Participants at lower risk for anxiety symptoms at baseline reported no significant change (and no escalation) in the GAD-7 scores at follow-up, on average, with 86.7% (255/291) maintaining their low symptom status. Participants at a lower risk for poorer well-being at baseline reported a small, significant improvement in the WHO-5 scores at follow-up, on average, with 90.1% (344/382) maintaining their low symptom status. Post hoc sensitivity analyses revealed that our models among lower-risk participants were sensitive to detect very small effect sizes (Cohen *d_z_*=0.16 for depressive symptoms, 0.16 for anxiety symptoms, and 0.14 for well-being), with 80% power and α=.05.

**Table 4:** Maintenance and change in mental health from baseline to follow-up among participants at lower risk at baseline (n=509).

Baseline symptoms^a^	Maintenance^b^, n (%)	Baseline, mean (SD)	Follow-up, mean (SD)	Cohen *d*	*t* test (*df*)	*P* value
Depressive symptoms (n=322)	297 (92.2)	4.82 (2.88)	4.39 (3.42)	0.13	2.26 (321)	.02
Anxiety symptoms (n=291)	255 (86.7)	3.79 (2.24)	4.00 (3.57)	−0.06	−1.01 (290)	.31
Well-being (n=382)	344 (90.1)	51.84 (14.07)	58.55 (18.83)	−0.40	−7.90 (381)	<.001

^a^Possible ranges for scores include 0-27 for depressive symptoms, 0-21 for anxiety symptoms, and 0-100 for well-being.

^b^Maintenance was indicated when participants did not meet or exceed the clinical cutoff at baseline or follow-up.

### Engagement in Recommended Care Plan

Of the 509 participants, 99 (19.4%) were recommended therapy and all lower-level services, 362 (71.1%) were recommended coaching and lower-level services, and 48 (9.4%) were recommended digital content only. Most participants (343/509, 67.4%) engaged with the level of care recommended to them; that is, they engaged at least once with their recommended care modality and did not step up above their recommended care. Specifically, of the 99 participants who were recommended therapy, 84 (85%) met with a therapist at least once; of the 362 participants who were recommended coaching, 224 (61.9%) met at least once with a coach; and of the 48 participants who were recommended digital content, 35 (73%) engaged with at least 1 piece of digital content.

A total of 16.9% (86/509) of the participants overused care beyond their original recommendation; that is, they used a care modality of higher intensity than they were recommended. Of the 362 participants who were recommended coaching, 73 (20.2%) intensified their care to meet with a therapist. Of the 48 participants who were recommended digital content, 13 (27%) intensified their care to access coaching and 2 (4%) participants intensified their care to access a therapist.

In total, 15.7% (80/509) of the participants underused care; that is, they did not engage with the care they were recommended or with a higher-intensity care. Specifically, 15 (15%) of the 99 participants who were recommended therapy declined the invitation to connect with a therapist, and 65 (10.8%) of the 362 participants who were recommended coaching declined the invitation to connect with a coach. A post hoc chi-square analysis comparing the likelihood of clinical improvement or recovery among groups of underusers, overusers, and those who engaged with their recommended level of care found no significant differences in outcomes between groups.

## Discussion

### Principal Findings

We examined the clinical effectiveness of and engagement in a digital mental health platform that uses a stratified blended care model to deliver mental health services. We found significant improvements in depressive, anxiety, and well-being symptoms among participants with elevated baseline symptoms and high rates of maintaining low symptoms and well-being among participants with lower clinical risk at baseline. Between 60% and 66% of the participants experienced clinically meaningful improvement or recovery in depressive, anxiety, or well-being symptoms over 3 months. We observed the greatest improvements in well-being (90% score increase), followed by depressive (37% score reduction) and anxiety symptoms (29% score reduction). These results are similar to the published rates of recovery in stepped care systems, which range from 40% to 60% [24].

Among participants with a lower baseline risk (defined as having scores that did not meet the clinical cutoff for that measure), mental health symptoms did not escalate above the clinical threshold or significantly worsen overall; anxiety symptoms remained stable, and depressive symptoms and well-being slightly improved on average. Between 87% and 92% of the participants experienced maintenance of good mental health at follow-up. Given that prevention and well-being promotion are cost-effective for mental health care and provide a positive return on investment for payers [25], our results lend further support to digital mental health services as being helpful for maintaining good mental health. Examining both symptom improvement and prevention of escalating symptoms is crucial to evaluating whether a model of mental health care is improving population health.

Most participants engaged with their personalized care recommendation, with only around one-third overusing or underusing services. Two-thirds (343/509, 67.4%) of the participants used the recommended level of services; that is, they engaged with their recommended care services but did not step up to use higher-intensity care. Rates of meeting the care recommendation were the highest for therapy, with 85% (84/99) of people who were recommended therapy having at least 1 visit with a therapist.

We also found that less than one-fifth (86/509, 16.9%) of the participants overused care; that is, they engaged with a care modality of a higher intensity than the one they were initially recommended. Although we did not have data available on referral pathways, participants could use a higher intensity of care through 2 channels: self-referral by contacting member services through the platform with their request and provider referral where a coach could refer a participant to a therapist if they had a demonstrated clinical need. Interestingly, patterns of overuse matched the intensity hierarchy of services: 20% (73/362) of the people who were recommended coaching moved up 1 level of intensity to therapy, 27% (13/48) of the people who were recommended digital content moved up 1 level to coaching, and only 4.2% (2/48) of the people who were recommended digital content moved up 2 levels to therapy. This further supports the accuracy of the initial care recommendations of this model. In addition, the fairly low rates of overuse are encouraging, considering long-held beliefs about psychotherapy as the gold standard for mental health treatment at all levels of care [26].

Finally, less than one-fifth (80/509, 15.7%) of the participants underused care; that is, they only engaged with lower-intensity care modalities than their recommendation. The rate was slightly higher for those who were recommended therapy (of the 99 participants, 15 (15%) did not have a visit with a therapist), while among those who were recommended coaching, 10.8% (65/362) did not have a visit with a coach or therapist. These rates of treatment nonadherence are consistent with those observed in psychotherapy research [27] and are better than those observed in previous research on digital interventions [28]. A post hoc analysis did not find statistically significant differences in improvement or recovery between people who followed their recommended care and those who overused or underused care. However, the lack of significance could be an artifact of low statistical power, given the skewed proportion of participants in the underuse and overuse categories. Future research that examines reasons for underusing or overusing care qualitatively would provide further insight into why this occurs for some people and may allow programs to stratify individuals into levels of care more appropriately.

It is difficult to compare the effectiveness of stratified blended care approaches in real-world contexts with that of other studies in the literature because most research has been conducted within highly controlled clinical trials, which lack ecological validity (eg, see Andrews et al [13] and Ho et al [29]). However, in one trial comparing stratified and progressive stepped care models, 76% of eligible screened patients engaged in treatment (high intensity or low intensity depending on personalized treatment recommendations) [16]. Thus, the engagement rate observed here is similar to that in research settings with high internal validity, suggesting that this platform is relatively accurate in recommending effective care. Our observational results of stratified blended care engagement patterns indicate that this model was efficient in matching individuals with the most effective and least costly care while also allowing them to self-determine their care and use combinations of services that best fit their needs.

### Limitations

Although this investigation demonstrated improvements in depression, anxiety, and well-being over time in a sample of digital mental health platform users, the observational nature of this study presents a limitation. The inclusion of a comparison or control group in an experimental design would confirm the causality of the observed changes. The 3-month time frame of this investigation also limits our conclusions to short-term gains; a longer-term follow-up period is needed to determine the persistence of improvements.

Only 9.25% (950/10,270) of the individuals who were eligible and invited to participate enrolled in this study. The reasons for this are not known but may be due to a combination of factors, such as the time commitment needed, as each survey took between 30 and 45 minutes to complete. In addition, although study materials highlighted the separation between the study and their employer, most invitations to participate were distributed to workplace email addresses, which may have given the impression to participants that their employers would be involved or aware of their participation in this investigation. Although the retention rate of this investigation was 73.26% (696/950) overall, the sample was limited to those who used at least 1 piece of content or sought a provider. More individuals were registered for the platform but did not use any care. This is common in real-world settings, as individuals may register for services without the intention of using them immediately. There were no significant differences on key demographic characteristics and baseline symptoms between the sample who met eligibility criteria and those who did not, which helps to bolster the generalizability of our findings.

In addition, we were unable to discern between self-referrals and provider referrals for the 16.9% (86/509) of the members who sought more intensive services beyond their initial care recommendation. It is possible that the 17% include some individuals who were appropriately identified for intensification of services (ie, a coach detected additional symptoms, or a member disclosed additional pertinent information that was not detected in the assessment algorithm that determines care recommendations).

Finally, most of the sample (312/509, 61.3%) identified as women, and only 34.4% (175/509) identified as men. Furthermore, the percentage of nonbinary people in this sample was higher than the overall US population [30] at 4.3%. There are several possibilities for the disproportionate numbers of women and nonbinary people enrolling in this study. First, the disproportionate number of nonbinary people in the sample may reflect a younger population, who are more likely to identify as transgender or nonbinary than older populations (5% vs 1.6% overall) [30]. In addition, we provided multiple nonbinary options for participants as opposed to a single all-encompassing option (eg, “nonbinary”), which may have encouraged identification. Finally, women and people who are lesbian, gay, bisexual, transgender, or questioning, including people who identify as nonbinary, are more likely to experience mental illness [31,32]. Women are more likely to seek help than men (due in part to societal expectations of stoicism and self-reliance for men, as well as mental health stigma), which might have affected their likelihood of signing up for the Modern Health app and participating in research [33]. Regardless of the reason for the lack of men in this sample, it is possible that the results here may be different among other populations, and we encourage future research to examine these potential differences.

### Conclusions

Overall, the results of this study lend support to the utility and effectiveness of the stratified blended care model used in this study to improve access to and use of mental health services. In a resource-constrained ecosystem, using a stratified blended model, such as the one evaluated, can make efficient use of limited and costly services while centering the individual’s needs, preferences, and receptivity to mental health care. The deliberate allocation of resources preserved the more intensive and costly resources for those who are most likely to benefit from them while providing beneficial care at all levels. As our results indicate, meaningful clinical improvements can be gained through stratified blended care while honoring the individual’s personal preference for how they want to engage in care.

Research continues throughout the field of mental health to determine the therapeutic approaches, techniques, and tools that can be adapted and disseminated for digital delivery while preserving safety, quality, validity, and efficacy [34]. As progress continues, the constraints of the traditional tertiary care model of mental health will eventually give way to a more comprehensive approach that can serve the full spectrum of mental health from primary prevention to treatment.

## Abbreviations

GAD-7: 7-item Generalized Anxiety Disorder scale
PHQ-9: 9-item Patient Health Questionnaire
WHO-5: 5-item World Health Organization Well-Being Index

## Data Availability

The data sets generated during and analyzed during this study are available from the corresponding author on reasonable request.
